# Stem cell modeling of nervous system tumors

**DOI:** 10.1242/dmm.050533

**Published:** 2024-02-14

**Authors:** Frank B. Furnari, Corina Anastasaki, Shan Bian, Howard A. Fine, Tomoyuki Koga, Lu Q. Le, Fausto J. Rodriguez, David H. Gutmann

**Affiliations:** ^1^ Department of Medicine, University of California, San Diego, San Diego, CA 92037, USA; ^2^ Department of Neurology, Washington University School of Medicine, St. Louis, MO 63110, USA; ^3^ Institute for Regenerative Medicine, School of Life Sciences and Technology, Tongji University, 200070 Shanghai, China; ^4^ Department of Neurology, Weill Cornell Medicine, New York, NY 10065, USA; ^5^ Department of Neurosurgery, University of Minnesota, Minneapolis, MN 55455, USA; ^6^ Department of Dermatology, University of Texas Southwestern Medical Center, Dallas, TX 75390, USA; ^7^ Division of Neuropathology, University of California, Los Angeles, Los Angeles, CA 90095, USA

**Keywords:** CRISPR engineering, Brain tumor, human embryonic stem cells (hESCs), human induced pluripotent stem cells (hiPSCs), Nerve sheath tumors

## Abstract

Nervous system tumors, particularly brain tumors, represent the most common tumors in children and one of the most lethal tumors in adults. Despite decades of research, there are few effective therapies for these cancers. Although human nervous system tumor cells and genetically engineered mouse models have served as excellent platforms for drug discovery and preclinical testing, they have limitations with respect to accurately recapitulating important aspects of the pathobiology of spontaneously arising human tumors. For this reason, attention has turned to the deployment of human stem cell engineering involving human embryonic or induced pluripotent stem cells, in which genetic alterations associated with nervous system cancers can be introduced. These stem cells can be used to create self-assembling three-dimensional cerebral organoids that preserve key features of the developing human brain. Moreover, stem cell-engineered lines are amenable to xenotransplantation into mice as a platform to investigate the tumor cell of origin, discover cancer evolutionary trajectories and identify therapeutic vulnerabilities. In this article, we review the current state of human stem cell models of nervous system tumors, discuss their advantages and disadvantages, and provide consensus recommendations for future research.

## Teaser

**Summary:** The application of human stem cell engineering to nervous system tumor modeling affords unique opportunities to study the cellular origins of tumors, examine cancer evolution and identify future therapeutic targets.

## Introduction

Cancer is one of the leading causes of death in children and adults, prompting the National Cancer Institute to launch the Cancer Moonshot initiative in 2016, with the goal of reducing cancer death, and improving the lives of people with cancer and cancer survivors. With the reinvigoration of this initiative in 2022 (Bertagnolli et al., 2023; Shiels et al., 2023), the development of therapeutic approaches to benefit those living with cancer has accelerated. One of the major areas of focus is increasing the pipeline of new cancer drugs and accelerating their translation to the clinical workplace. To address this challenge, there is a pressing need to develop platforms amenable to rapid drug screening and therapeutic validation. In the past, lead compounds were identified using immortalized cancer cell lines maintained in standard tissue culture medium (Gazdar et al., 2010). Although this approach has resulted in the identification of numerous therapeutic anti-cancer compounds, the tumor lines employed do not resemble their native parental counterparts. As such, immortalized cancer cell lines lack dependence on stroma (Box 1), consisting of non-neoplastic cells in the tumor microenvironment, and often acquire additional genetic or molecular changes during hundreds or thousands of *in vitro* passages (Kim et al., 2017; Sinha et al., 2021). Similarly, patient tumor-derived cell lines lose the heterogeneity and the genomic, transcriptomic and epigenetic signatures of their respective original cancer (Li and Langhans, 2021) and, in the case of less malignant tumors (e.g. pilocytic astrocytoma and neurofibroma) (Box 1), they undergo oncogene-induced senescence or fail to propagate after several passages (Esparza-Lopez et al., 2019; Yuan et al., 2021).

**Box 1. Glossary**

**Astrocytes:** Type of glial cell in the central nervous system.

**Astrocytoma:** Brain tumor originating from astrocytes, i.e. star-shaped glial cells of the brain and spinal cord.

**Cancer stem cells (CSCs):** Subpopulation of cells within tumors, harboring stem cell properties.

**Choroid plexus:** Structure containing ependymal cells and capillaries that secrete and circulate cerebrospinal fluid.

**Chromosomal copy number variants (CNVs):** Alterations in chromosome numbers.

**Cortical layer:** Outer layer of neural tissue in the brain.

**CRISPR-C:** Clustered regularly interspaced short palindromic repeats (CRISPR) editing method to create extrachromosomal circular DNA.

**Ependymal cells:** Ciliated neuroepithelial cells lining the CNS ventricles.

**Ependymoma:** Glial tumor cell growth arising from ependymal cells.

**Forebrain:** Largest and most highly developed part of the brain.

**Glial cell:** Non-neuronal cell in the central nervous system.

**Glial-restricted progenitors:** Progenitor cells that can generate astrocytes.

**Glioma cerebral organoid (GLICO) model:** *Ex vivo* brain tumor model consisting of pluripotent stem cell-derived organoids mixed with patient tumor cells.

**Glioblastoma (GBM):** Grade 4 astrocytoma.

**Glioma:** Astrocytic tumor of the central nervous system.

**Glioma stem cells (GSCs):** Brain tumor cells with self-renewal and differentiation capacity.

**Human cerebral organoids (hCOs):** Human brain organoids that can develop tumors by introducing tumorigenic mutations into normal PSCs.

**Medulloblastoma:** Malignant brain tumor arising in the cerebellum.

**Meninges:** Membranous tissue enclosing the brain and spinal cord.

**Meningioma:** Tumor arising from meningeal cells.

**Microglia:** Monocyte population unique to the brain.

**Nerve plexus:** Network of intersecting nerves.

**Neuroepithelial ventricular zones:** Brain areas containing ependymal cells.

**Neurofibroma:** Benign peripheral nerve sheath tumor.

**Oligodendrocytes:** Glial cells of the central nervous system, capable of ensheathing neurons with myelin.

**Organoid:** Tissue-engineered cell-based organ model.

**Pilocytic astrocytoma (PA):** Grade 1 astrocytoma.

**Pluripotent stem cells (PSCs):** Stem cells that have the ability to self-renew and differentiate into all cell types within tissues of the body.

**Radial glia:** Specialized glial cells with long radial processes.

**Schwann cells:** Glial cells of the peripheral nervous system, capable of ensheathing neurons with myelin.

**Schwannoma:** Schwann cell tumor.

**Stroma:** Tumor tissue comprising non-neoplastic cells.

**Stromal dependence:** Inherent requirement for stromal cell support of tumor growth.

**Teratoma:** Germ cell tumor comprising immature or fully formed tissues.

By contrast, genetically engineered mouse models of human cancers do have the ability to faithfully recapitulate stromal dependencies, tumor heterogeneity and developmental contexts that typify the parental tumors (Marks, 2009). However, they are not efficient models for drug discovery or high-volume preclinical drug testing and only occasionally capture the unique aspects of the human tumors (Tomita et al., 2022), including their cellular origins, tissue and cellular heterogeneity, mutational evolution, heterogeneous chromosomal copy number variants (CNVs) (Box 1), drug metabolism and/or stromal cell composition. Additionally, certain genetic alterations found in human tumors are not easily modeled in the mouse. A prime example of this is the telomerase reverse transcriptase (TERT) promoter mutation, which cannot be recreated in mouse somatic cells due to their – relative to humans – long telomere regions and divergent promoter sequence, (Miki et al., 2022; Zhang et al., 2016). For these reasons, an emphasis has recently been placed on the deployment of human stem cell engineering to develop robust *in vitro*, *ex vivo* and *in vivo* models suitable for therapeutic drug discovery and evaluation (Khamis et al., 2023; Liu et al., 2016; Weth et al., 2022).

The use of human stem cell engineering to study cancers has particular relevance for tumors of the central and peripheral nervous systems. These tumors comprise a wide variety of histologic types and malignancy grades (Horbinski et al., 2022; Pellerino et al., 2023). Specifically, central nervous system (CNS) tumors include low-grade and high-grade neoplasms of the brain (glioma, medulloblastoma and ependymoma) (Box 1), meninges (meningioma) (Box 1), and spinal cord (ependymoma and glioma), while those affecting the peripheral nervous system (PNS) include neurofibromas and schwannomas (Box 1). For all of these nervous system tumors, there is a paucity of humanized models and few efficacious treatments. In this article, as leaders in the field of human stem cell nervous system engineering, we detail the current state of the field, the associated advantages and disadvantages of various stem cell-based models and the opportunities for further refinement relevant to CNS and PNS tumors.

## Human stem cell engineering

**Fig. 1.**
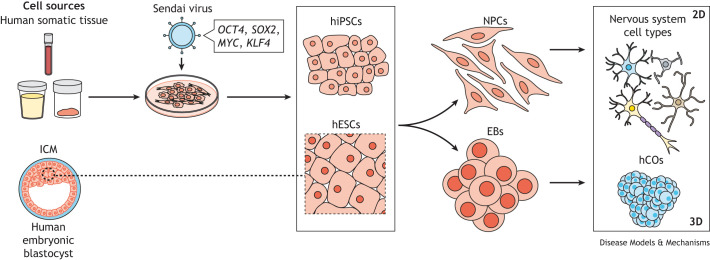
**Generating 2D and 3D models of nervous system cells from human tissues.** Human induced pluripotent stem cells (hiPSCs) can be generated from somatic tissue (e.g. blood, urine, skin biopsies), by expressing the four Yamanaka transcription factor genes (*KLF4*, *OCT4*, *MYC* and *SOX2*) using Sendai virus. Alternatively, human embryonic stem cells (hESCs), derived from the inner cell mass (ICM) of human embryonic blastocysts, can be isolated. Both can be used to generate neural progenitor cells (NPCs) or embryoid bodies (EBs), which can give rise to cell types of the nervous system (e.g. neurons, astrocytes, oligodendrocytes) in 2D culture or to human cerebral organoids (hCOs) in 3D culture. Microglia and Schwann cells can also be produced using specific medium and additives that specify differentiation into monocyte and peripheral myelinating glia cell lineages, respectively.

Human stem cells can be isolated from human embryonic tissues from the inner cell mass of blastocyst-stage (or earlier) embryos (Peura et al., 2011; Thomson et al., 1998). Alternatively, somatic tissues (e.g. skin, blood and epithelial cells) can be reprogrammed into pluripotent stem cells (PSCs) (Box 1) using a cocktail of transcription factors called Yamanaka factors (OCT4, SOX2, KLF4 and MYC), named after Shinya Yamanaka, who was awarded the 2012 Nobel Prize for this groundbreaking technology (Takahashi et al., 2007; Takahashi and Yamanaka, 2006). These factors are typically introduced using a Sendai virus vector, which does not integrate into the host genome. A host of other techniques for introducing the obligate mRNA and/or proteins have also been successfully used (reviewed by Kim et al., 2011). The resulting PSCs can be differentiated into a myriad of neuroglial cell (Box 1) lineages that can be grown as: (i) two-dimensional (2D) monolayers, including astrocytes, Schwann cells, glial-restricted progenitors, oligodendrocytes and various neuronal cell populations (Box 1); (ii) three-dimensional (3D) spheroids containing self-assembling brain-region-specific organoids (e.g. cortical); (iii) and cerebral organoids (Lancaster and Knoblich, 2014; Lancaster et al., 2013) (Box 1) representing different brain regional identities (Whye et al., 2022) (Fig. 1). Similar to the developing human brain, the most neuroanatomically mature one of these models, i.e. the cerebral organoid, contains germinal zones that give rise to neurons, astrocytes and oligodendrocytes in a time-dependent manner, and can be maintained for more than one year *in vitro* (Giandomenico et al., 2021).

A powerful use of these PSC models is that they can be genetically manipulated to introduce cancer-associated insertions, deletions and single nucleotide mutations into specific genomic sequences. This is most typically accomplished by using CRISPR/Cas9 technology (Byrne and Church, 2015; Mali et al., 2013), although other methods have also been used, including TALEN-based engineering (Ding et al., 2013), RNA-based interference (Ivanova et al., 2006) or zinc finger nuclease-based engineering (Ding et al., 2013; Hockemeyer et al., 2009; Ivanova et al., 2006; Liu et al., 2011; Yusa et al., 2011; Zou et al., 2009). The ability to generate specific neuroglial cell populations harboring cancer-causing genetic alterations in either 2D or 3D configurations offers unprecedented opportunities to study the cellular origins of brain and peripheral nerve tumors, the impact of specific genomic alterations on tumorigenesis, and the interactions between neoplastic cells and their non-neoplastic cell neighbors.

## Stem cell modeling of nervous system tumors

**Fig. 2.**
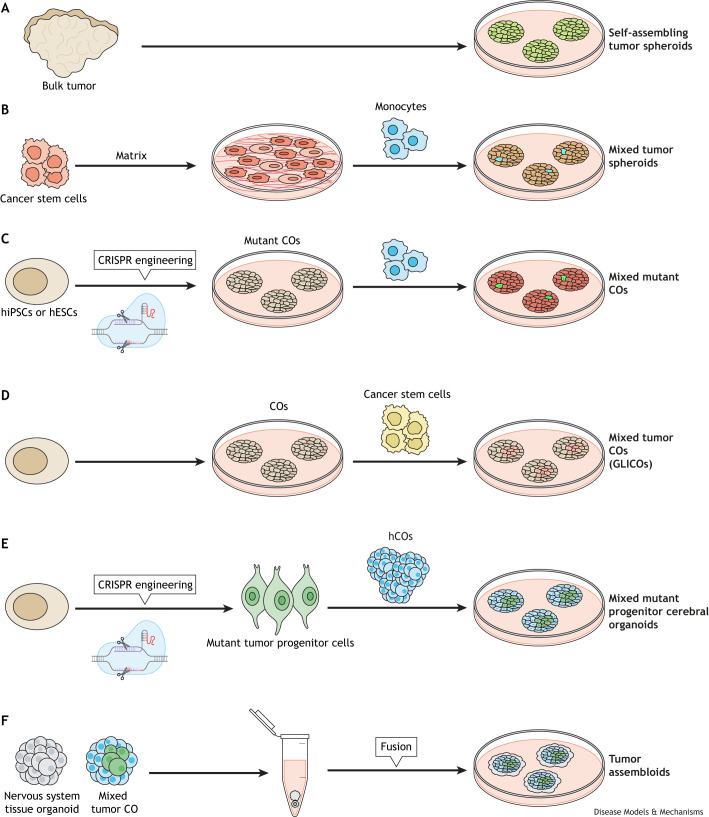
***In vitro* approaches for modeling nervous system tumors.** (A) Self-assembling tumor spheroids can be grown from dissociated bulk tumor specimens. (B) Cancer stem cells can be grown in defined matrix (Matrigel) with the addition of monocytes (microglia or macrophages) to generate mixed tumor spheroids. (C) Human induced pluripotent stem cells (hiPSCs) or embryonic stem cells (hESCs) can be CRISPR engineered to harbor mutations associated with cancer of the nervous system, yielding mutant cerebral organoids (COs), as well as mixed COs following the addition of monocytes (or other cell types). (D) hiPSCs or hESCs induced to form cerebral organoids (COs) can be mixed with cancer cells to form mixed tumor cerebral organoid gliomas (GLICOs). (E) Tumor progenitor cells generated from hiPSCs or hESCs carrying mutations associated with cancer of the nervous system can be mixed with hCOs to produce mixed mutant progenitor COs to model tumor evolution. (F) Tumor assembloids containing multiple nervous system tissues can be created by fusing mixed tumor CO with nervous system tissue organoid representing other tissues, such as optic nerve, spinal cord or brainstem.

The use of human stem cells to model nervous system tumors involves several approaches, including (i) the generation of primary differentiated cell types harboring cancer-associated mutations for analysis in 2D culture (Anastasaki et al., 2022; Modrek et al., 2018); (ii) the integration of stem cell-derived cell types, like microglia (Box 1), into mouse organotypic culture systems (Huang et al., 2022) and; (iii) the use of brain tumor organoids. These latter organoid models, derived either from patient tumor specimens or PSCs, not only faithfully recapitulate important aspects of these tumors, but also provide innovative platforms for conducting preclinical investigations into tumor treatment strategies (Fig. 2). For a summary of the models discussed in this article, see Table 1.

**Table 1.**
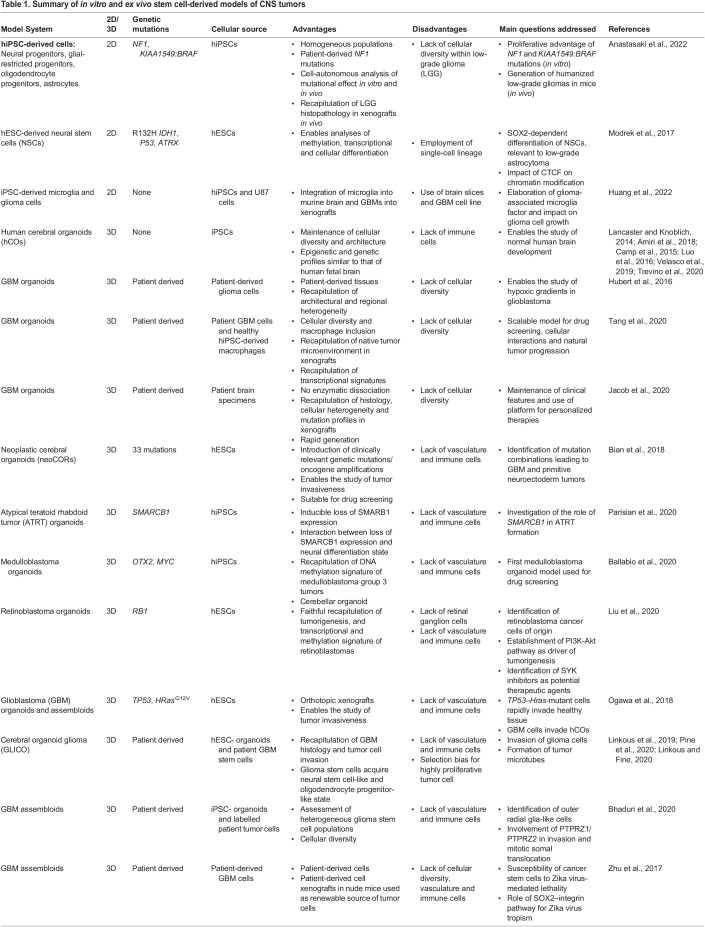
Summary of *in vitro* and *ex vivo* stem cell-derived models of CNS tumors

### *In vitro* organoid and assembloid models

The term ‘organoid’ was first used 40 years ago to describe tumors with complex tissue-like structures (Nesland et al., 1985). However, the ability to establish 3D tissue-like tumor cultures *in vitro*, by using either cancer stem cells (CSCs) (Box 1) or patient tumor biopsies, has only recently become possible. CSCs are a rare population of cells within tumors known for their stem cell properties, including the ability to self-renew and differentiate into various types of nervous system cell (Singh et al., 2004). By embedding CSCs isolated from malignant brain tumor specimens, such as glioblastoma (GBM), into scaffold materials (e.g. Matrigel), Hubert and colleagues established a 3D culture system capable of supporting long-term expansion that replicates many key aspects of the original tumors (Hubert et al., 2016). These GBM organoids (GBOs) recapitulate the architectural characteristics, regional tumor heterogeneity and low oxygen gradients found in the parental tumor tissues. To better reproduce the native tumor microenvironment *in vivo*, the same research group introduced macrophages into a CSC-based bioprinted 3D culture system, a tissue-engineering platform where cells are incorporated within hydrogels that contain biodegradable polymers. They found that these macrophage-integrated GBM-bioprinted models more closely resemble the transcriptional signatures that typify the original tumors (Tang et al., 2020). Brain tumor organoids can also be directly established from patient brain specimens, which may contain CSCs. For example, a GBO culture platform was developed from parental tumor tissues without the need for mechanical or enzymatic dissociation (Jacob et al., 2020). These patient specimen-derived GBOs faithfully recapitulated the histological features, cellular heterogeneity and mutation profiles of the original tumors. Owing to their rapid production and high reliability, this GBO model is well-suited for large-scale biobanking. These patient-derived brain tumor organoid models, whether derived from CSCs or biopsies, can provide excellent platforms for future personalized medicine applications.

Alternatively, brain tumor organoids can be developed by introducing tumorigenic mutations into normal PSCs, as they generate human cerebral organoids (hCOs) (Fig. 1, Box 1) (Lancaster et al., 2013). These 3D cell culture models mimic *in vivo* cellular diversity and structures of multiple brain regions, and share similar transcriptomic, proteomic and epigenetic profiles with human fetal brains (Amiri et al., 2018; Camp et al., 2015; Luo et al., 2016; Trevino et al., 2020; Velasco et al., 2019). Consequently, hCOs have widespread use for studying human brain development, evolution and modeling brain diseases, including brain tumors (Di Lullo and Kriegstein, 2017). For example, Bian and colleagues analyzed tumor formation in human brain organoid cultures by introducing clinically relevant genetic mutations and/or oncogene amplifications using transposon/CRISPR-Cas9 editing (Bian et al., 2018). This approach allowed for the generation of two distinct brain tumor subtypes, GBM and central nervous system embryonal tumors. These brain tumor organoid models exhibit similar transcriptional signatures, cellular heterogeneity and invasive characteristics as their parental tumors *in vivo*. In another study, Ogawa and colleagues adopted a similar genetic manipulation approach to create a GBM organoid model by disrupting *TP53* and activating *HRas^G12V^* (Ogawa et al., 2018), while Parisian and colleagues established an organoid model of atypical teratoid rhabdoid tumors (ATRTs), a pediatric brain cancer, in hCOs by inactivating the *SMARCB1*, a gene important for chromatin remodeling (Parisian et al., 2020).

As various region-specific brain organoid models have been developed by introducing patterning factors into the culture, region-specific tumor organoids have also emerged (Qian et al., 2018; Sloan et al., 2018; Xiang et al., 2017). For example, a medulloblastoma organoid model was established by overexpressing *OTX2* and *MYC* in human cerebellar organoids to replicate the features of medulloblastoma group 3 tumors (Ballabio et al., 2020). Similarly, Liu and colleagues generated a retinoblastoma organoid model by culturing retinal organoids, using human embryonic stem cells (hESCs) carrying homozygous mutations in the *RB1* gene to drive tumorigenesis (Liu et al., 2020). By using this model, they identified ARR3-positive cone precursor cells as the origin of retinoblastoma cells and PI3K/AKT pathway hyperactivation as the driver of retinoblastoma tumor cell growth, and discovered that inhibition of the spleen tyrosine-protein kinase (SYK) reduced retinoblastoma cell proliferation.

### *Ex vivo* GLICO models

To investigate interactions between tumor tissues and normal brain tissues, as well as the invasive features of brain tumors, researchers have developed tumor assembloid models. These models involve fusing PSC-derived brain organoids with either tumor cells obtained from patient specimens, termed glioma cerebral organoid (GLICO) model (Linkous et al., 2019) (Box 1), or with tumor cells generated through genetic manipulation of PSCs (Ogawa et al., 2018). In these models, the brain organoids represent the normal brain microenvironment, facilitating the discovery that GBM tumor cells deeply invade the normal organoid parenchyma by forming interconnected networks of tumor microtubes (Linkous et al., 2019). Additionally, Bhaduri and colleagues identified outer radial glial (oRG)-like cells (Box 1) as an invasive subtype of tumor cell (Bhaduri et al., 2020), which invaded brain organoids after engraftment, suggesting that this subpopulation of tumor cells plays a crucial role in tumor progression. Demonstrating the clinical translatability of such models, another group used GBM assembloids to identify the SOX2–integrin axis as a potential mechanism for Zika virus tropism – a potential oncolytic viral therapeutic strategy in GBM (Zhu et al., 2017).

To produce GLICOs, glioma stem cells (GSCs) (Box 1) are grown *ex vivo* within hCOs derived from either generic hESCs or autologous patient-derived iPSCs. The resulting hCOs closely mimic the neurodevelopmental trajectory of the human CNS from the early embryonic period through the first year of life (Eichmuller and Knoblich, 2022). hCOs have predominantly forebrain (Box 1) identity, as evidenced by the presence of an underlying neuroepithelial ventricular zone with a cortical layer (Box 1) along with a vast array of diverse brain cell types, including neural stem cells, radial glia, neuronal and glial subtypes, as well as other neuroepithelial-derived components (e.g. retinal pigmented epithelia, choroid plexus and ependymal cells) (Box 1). Single-cell RNA sequencing data has revealed that the GSCs isolated from GLICO models more closely recapitulate the diverse distribution of cellular transcriptomic states found in the patient's own GBM (Pine et al., 2020). In particular, GLICO appears to more readily allow GSCs to acquire their neural stem cell-like (NSC-L) and oligodendroglioma progenitor-like (OPC-L) states, in contrast to the more differentiated mesenchymal-like (MES-L) and astrocytic-like (AC-L) states, which appear to be dominated by the stress-associated MES-L state (Pine et al., 2020).

In addition, using single-cell ATAC sequencing coupled with multi-omics data, the GLICO model has allowed for the deconvolution of the epigenetic foundations and chromatin dynamics of the GSC and/or GBM cellular states, highlighting similarities to normal human early neurodevelopment (Pine et al., 2023). Moreover, early data suggest that these differential transcriptomic states, found in patients and reproduced in GLICO, translate to differential therapeutic drug sensitivities in GLICO relative to other model systems (Linkous et al., 2019). Whether or not these different drug sensitivities are more predictive of clinical drug sensitives in a patient-specific manner is currently being experimentally explored.

Although GLICO has proven to be a useful patient-specific and tractable model for studying multiple aspects of GBM, it – like all models – has clear limitations. One of the greatest limitations results from the fact that brain vasculature is derived from mesodermal tissues. Thus, the GLICO model lacks endothelial cells, is avascular and, therefore, does not model the blood–brain barrier (BBB). Additionally, the GLICO model neither has an extrinsic immunologic niche (e.g. lymphocytes) nor an intrinsic one, as microglia are derived from embryonic yok sac mesodermal components (Ginhoux et al., 2010). Finally, the current state of bioengineering of cerebral organoids does not reproduce the spatial representations of the spontaneously forming cerebrum. For this reason, if there are specific regional/spatial microenvironmental effects on GSCs within the brain, it might be difficult to reliably model this with the current state of the technology.

Future efforts to improve the GLICO model include the incorporation of ever-improving technologies for more sophisticated stem cell-derived COs, the development of novel ways to vascularize GLICO models (e.g. with endothelial cells that closely recapitulate the BBB) and incorporation of stem-cell derived microglia in COs, thereby producing an autologous *ex vivo* patient-specific immunological niche (Abud et al., 2017). With these refinements, *ex vivo* GBM models, such as GLICO, may offer sophisticated solutions to uncover novel glioma biology and to develop and screen effective new therapeutics in a patient-specific manner.

## *In vivo* tumor models

### Peripheral nerve sheath tumor models

Nerve sheath tumors are, typically, benign tumors that can develop on or in the peripheral nerves at any location in the body. Although benign, these tumors can press against nerves, causing pain or numbness and, ultimately, damage that leads to loss of nerve function (Schulz et al., 2018). They are often associated with neurofibromatosis (NF), a tumor predisposition condition with three distinct genetic subgroups: neurofibromatosis type 1 (NF1), neurofibromatosis type 2 (NF2)-related schwannomatosis and non-NF2-related schwannomatosis (SWN). Individuals diagnosed with NF1 have pathogenic variations in the *NF1* gene, which encodes neurofibromin 1, a large RAS GTPase activating protein (Bergoug et al., 2020). These patients develop a variety of clinical manifestations including neurofibromas, benign nerve sheath tumors that can develop in the dermis along cutaneous nerve twigs (cutaneous neurofibroma) (Liao et al., 2016) or deeper in the body within a nerve plexus (plexiform neurofibroma) (Box 1) (Rodriguez et al., 2012). Individuals with pathogenic changes in the *NF2* gene, which encodes merlin, a cytoskeletal linker protein that regulates Hippo–YAP signaling (Petrilli and Fernandez-Valle, 2016), can develop benign nerve sheath tumors called schwannomas anywhere in the body. However, the hallmark manifestation of NF2 is development of a bilateral vestibular schwannoma (VS) that develops in the auditory vestibular (also known as vestibulocochlear nerve or cranial nerve VIII) nerve, often leading to deafness (Evans, 2009). SWN has a phenotypic overlap with NF2, characterized by the presence of schwannomas, but without VS development (MacCollin et al., 2005) and with distinct underlying genetics (Plotkin et al., 2022), including mutations identified in *SMARCB1* and *LZTR1* (Hulsebos et al., 2007; Piotrowski et al., 2014).

**Fig. 3.**
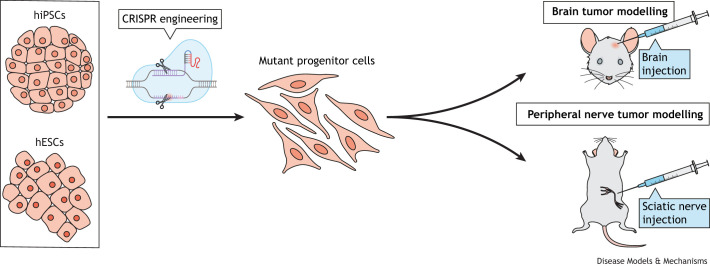
***In vivo* modeling of CNS tumors.** Human induced pluripotent stem cells (hiPSCs) or embryonic stem cells (hESCs) can be used to generate mutant progenitor cells harboring mutations associated with cancer of the nervous system by CRISPR engineering. These mutant progenitor cells can then be injected into the brain or sciatic nerve of immunocompromised rodents for brain tumor or peripheral nerve tumor modeling studies, respectively.

Although several mouse models for NF1-associated neurofibromas and NF2-associated schwannomas have been developed and successfully used to explore tumor biology and evaluate promising therapies (Chen et al., 2019; Gehlhausen et al., 2015; Giovannini et al., 2000; Mayes et al., 2011; Wu et al., 2008; Zheng et al., 2008; Zhu et al., 2002), PSC engineering offers the opportunity to generate humanized mouse models of these tumors. In one study, iPSCs engineered to harbor specific *NF1* mutations (Anastasaki et al., 2020; Mo et al., 2021) were differentiated into Schwann cell precursors and implanted near the sciatic nerve of athymic nude mice to form plexiform neurofibromas (Mo et al., 2021). When these cells were further engineered to harbor loss of *TP53*, they developed malignant peripheral nerve sheath tumors in mice (Mo et al., 2021). Similarly, *NF1−/−* spheroids generated *in vitro* from iPSCs and then implanted near the sciatic nerve in athymic mice also formed plexiform neurofibromas (Mazuelas et al., 2022). These humanized models establish robust platforms to enable mechanistic studies on the phenotypic consequences of particular *NF1* mutations as well as drug screening (Fig. 3).

### Low-grade brain tumor models

hiPSCs have also been employed to develop models of pediatric low-grade glioma (pLGGs; grade 1 and 2 gliomas) *in vitro* (Modrek et al., 2017) and *in vivo* (Anastasaki et al., 2022). Leveraging stem cell technology is imperative for understanding LGG gliomagenesis and progression because of the relative paucity of patient-derived surgical specimens and xenograft models, as well as their propensity to undergo cellular senescence (Buhl et al., 2019; Jacob et al., 2011).

In addition, mimicking the two most common genomic alterations seen in pLGGs – specifically pilocytic astrocytomas (PAs) classified as grade 1, non-malignant tumors of the CNS by the World Health Organization (WHO) – hiPSCs were engineered to harbor bi-allelic *NF1* mutations or ectopic expression of *KIAA1549: BRAF* (Anastasaki et al., 2022). Given their inherent ability to differentiate into all brain cell lineages, these hiPSCs were then employed to generate LGGs that are histologically similar to their human counterparts (Fig. 3). These cells helped establish that the likely cells of origin for pLGGs were glial-restricted progenitors or oligodendrocyte progenitor cells. It was possible to maintain these hiPSC-derived LGGs as xenografts for at least six months *in vivo* without any notable effects on mouse health and to visualize them by using small-animal magnetic resonance imaging (MRI). As such, they were employed to demonstrate that inhibition of mitogen-activated protein kinase (MAPK) signaling, a hallmark of pLGGs, was sufficient to attenuate tumor growth (Anastasaki et al., 2022).

## hiPSC platforms for functional genomics and pharmacological drug screens

As iPSC-derived models have become more reproducible and robust, they are now being employed in large-scale drug discovery and functional genomics efforts. The combination of CRISPR knock-out (KO) and knock-in (KI) screens, coupled with improved iPSC-derived models, has begun to elucidate underlying pathogenic mechanisms and guide therapeutic strategies for nervous system tumors. Additionally, tools – such as CRISPR interference (CRISPRi) and activation (CRISPRa) – provide complementary ways to alter the expression of endogenous genes (Konermann et al., 2015; Larson et al., 2013) and identify transcription factors required for hiPSC brain cell function. As shown by Chavez and colleagues, a pool of guide RNAs (gRNAs) recognizing the pro-neural factor genes *NGN2* or *NEUROD1* promotes iPSC differentiation into neurons (Chavez et al., 2015). Given the theme of arrested neuronal development in pediatric brain cancers, such as ATRTs (Parisian et al., 2020), diffuse midline gliomas (Jessa et al., 2019) and medulloblastomas (Hendrikse et al., 2022; Smith et al., 2022), similar screening strategies to identify pathways that promote terminal differentiation might emerge as viable approaches to discover novel therapies (Black et al., 2020). Although drug screens using iPSC-derived models and recapitulating cardiomyocyte (Weng et al., 2020), hepatocyte (Cayo et al., 2017), neuronal (Young et al., 2018) and neural progenitor (De Masi et al., 2020; Readhead et al., 2018) cell types have been successful, such screens are yet to be conducted on iPSC-derived tumor models of the nervous system.

## Factors to consider for stem cell-derived models

With the advent of iPSC technologies, we now have ample customizable methods to simulate cancers of the nervous system *in vitro* and *in vivo.* However, several important parameters need to be considered when developing stem cell-derived models. First, due to phenotypic variation between lines, will the chosen stem cell line make it challenging to replicate findings in different labs? Second, has the reprograming strategy led to genomic alterations, such as chromosomal aneuploidy, CNVs and/or point mutations, which decrease genetic integrity (reviewed by Poetsch et al., 2022) and influence disease modeling accuracy? Addressing these concerns, a recent study assessed nine widely used hiPSC lines by using whole-genome sequencing to detect genome stability post hiPSC reprogramming and post gene editing, and ability to differentiate into commonly used cell types. Strikingly, only one line, i.e. KOLF2.1J, performed well when using a range of differentiation protocols and functional assays (Pantazis et al., 2022).

Equally important is the development of a reproducible and scalable differentiation protocol for each desired cell type. Establishment of gold-standard differentiation parameters, e.g. expression of cell marker or profiling cellular functions, is necessary to ensure the faithful recapitulation of cell type phenotypes and avoid insufficient differentiation that could lead to the formation of teratoma (see Glossary) following engraftment in mice (Koga et al., 2020; Reinhardt et al., 2013). Similarly, it is important to consider somatic epigenetic memory, reflected in incompletely reset DNA methylation patterns within partially reprogrammed hiPSCs (Maherali et al., 2007; Mikkelsen et al., 2007), which might influence the differentiation potential of the stem cell lineage and their utility for drug screening and accurate disease modeling (Ohi et al., 2011). While hiPSC-derived CNS tumors can recapitulate transcriptional signatures of patient tumors (Koga et al., 2020; Parisian et al., 2020), it is unclear how well their DNA methylation patterns reflect tumor-specific profiles used for tumor classification (Haag et al., 2021). This is an area for further investigation and should be considered if hiPSCs are partially reprogrammed; it would be interesting to determine whether hESCs better recapitulate tumor-specific DNA methylation profiles when used for model development. Additionally, a direct comparison with iPSCs generated from patient tumors would help determine whether the DNA methylation programs indicative of tumor type are maintained in the reprogrammed state (Kong et al., 2020).

## Credentialing of models

Although no tumor model will recapitulate all of the features of their native human counterparts, it is important to authenticate the platform by using established neuropathological criteria and take into account the responsible genetic driver mutations. The standard for brain tumor diagnosis is the 2021 WHO classification of central nervous system tumors (Louis et al., 2021). In the case of gliomas, the predominant primary brain tumor throughout lifespan, low- and high-grade diffuse gliomas are separated by the age group predominantly affected (pediatric-type versus adult-type gliomas), as well as their underlying genetic drivers. For example, adult gliomas are often driven by *IDH1* or *IDH2* mutations (Cancer Genome Atlas Research Network et al., 2015), while subsets of low-grade pediatric gliomas are driven by rearrangements in the transcription factor-encoding proto-oncogenes *MYB* or *MYB* like 1 (*MYBL1*) (Qaddoumi et al., 2016). Meanwhile, high-grade pediatric-type gliomas are more commonly associated with histone mutations (Schwartzentruber et al., 2012).

When validating brain tumor models, it is important to examine the specific pattern of growth at the histological level. In this regard, diffuse high-grade gliomas (CNS gliomas at WHO grades 3 or 4) are characterized by variable combinations of high cellularity, increased mitotic activity, microvascular proliferation and necrosis, with exquisite infiltration of the underlying brain parenchyma (Berger et al., 2022). In contrast, circumscribed gliomas have a more compact architecture. These histological features should be present in the resulting models as judged by an experienced neuropathologist (Vaubel et al., 2020). However, it is important to recognize that an increasing number of specific brain tumor types are now defined by their underlying genetic drivers, even when histologic features are ambiguous. For this reason, histological grading of preclinical tumor models independently of genetic driver context is not advisable.

In addition to histological criteria, phenotypic characterization using relevant markers of differentiation, including glial (e.g. GFAP, OLIG2 and S100) and neuronal (e.g. synaptophysin) immunostaining, is essential (Louis et al., 2021). Additionally, as a surrogate of total cellular biology and function, transcriptomic signatures of any given human brain tumor model should be highly reflective of the transcriptome of the corresponding primary human tumor. Similarly, methylation profiling has demonstrated great clinical utility in classifying many human brain tumors, facilitating the identification of clinically relevant subtypes (Capper et al., 2018). Although it is expected – as indicated above – that methylation-based classification will be useful for validating brain tumor models, its utility in this context at the present time, awaits further investigation.

Similarly to brain tumors, peripheral nerve tumor models should be validated using standard histological, phenotypic and genetic analyses. This evaluation includes routine histological examination and immunophenotypic characterization using appropriate cell type-specific antibodies (Tekavec et al., 2022). Additionally, electron microscopy has great utility for the analysis of peripheral nerve tumor models, since the various cell components of the peripheral nerve sheath have unique ultrastructural properties and their architectural relationship with the parent nerve can be assessed at the ultrastructural level (Wu et al., 2008).

Finally, as observed in patients with these tumors, it is important to consider the impact of the tumor on brain and nerve anatomy and function. In this manner, MRI is often deployed to characterize the relationship between tumors and their surrounding nervous system environment, while electrodiagnostic tests, like nerve conduction and electroencephalography evaluations, could be used to reveal functional consequences of the tumor on their associated nerves or brain. Similarly, analysis of rodents with these tumors for clinical features typically seen in affected people (e.g. vision loss, leg weakness and hydrocephalus), should be performed and their effects on survival carefully recorded.

## Challenges and future directions

With the advancement of genome engineering technologies and stem cell reprogramming, it has become feasible to introduce bona fide genetic alterations observed in patient nervous system tumors into the presumed tumor cells of origin. This can both recapitulate the authentic pathobiology of patient tumors (Anastasaki et al., 2022; Koga et al., 2020) and enable the investigation of the primary function of specific mutations in a cell-autonomous, isogenic fashion. In addition, it can lead to the identification of targetable vulnerabilities of specific cell types that are susceptible to the engineered genetic alterations. Particularly when applied in the context of synthetic lethality, methods such as CRISPR/Cas12 editing (Kleinstiver et al., 2019), can potentially accelerate the development of novel effective therapeutics for nervous system tumors. Despite the benefits of the multitude of emerging stem cell-derived preclinical models to study the development, progression and treatment of nervous system tumors, there are still significant challenges to overcome.

First, the genetic complexity and heterogeneity of the tumor microenvironment is not always accurately depicted using existing methods. For instance, many nervous system tumors, including GBM and medulloblastoma, are driven by dominant oncogene amplifications on extrachromosomal circular DNA (Turner et al., 2017), the engineering of which remains challenging. Targeted circularization of particular oncogene loci by using CRISPR-C (Moller et al., 2018) (Box 1) may enable incorporation of hallmark genetic alterations of those nervous system tumors in future models. Additionally, the inherent genetic heterogeneity of some gliomas, partly owing to the multitude of different cell types within the tumor microenvironment, has not been modeled by current preclinical models. Future stem cell-derived models could provide a more faithful landscape of these common brain cancers by encompassing tumor cells that harbor multiple concomitant mutations or by temporally controlling the expression of multiple genetic alterations in combination with non-neoplastic cells. This could be achieved by distinct methods, such as 3D bioprinting of iPSC-derived models with brain microenvironment components (Tang et al., 2020), incorporation into the GLICO platform or by using a xenotransplantation platform consisting of human brain organoids seeded with tumor cells and microenvironment cell constituents (e.g. microglia) that are engrafted in immunocompromised mice (Mansour et al., 2018; Schafer et al., 2023), to better recapitulate transcriptional programs that depend on the human brain environment. To overcome heterogeneity issues associated with population differences (e.g. ethnicity and sex), future models could use multiple clones from cell line panels that capture differences in background genetics, such as those presented in the Human Induced Pluripotent Stem Cells Initiative (HipSci) (Kilpinen et al., 2017).

Second, hESCs and hiPSCs provide unique opportunities to generate multiple cell types to examine putative tumor cells of origin. However, specific cell lineages are intimately associated with different methylation profiles and epigenetic changes are, thus, essential features shaping each specific cell type, including varieties of central nervous system tumor cells (Mackay et al., 2017). Correct selection of the putative cell of origin is crucial in recapitulating such epigenetic alterations (Haag et al., 2021), and future studies could target an expanded cellular population for the modeling of each tumor type. Alternatively, epigenome modification engineering (Takahashi et al., 2017), in combination with evolving technologies in cellular differentiation of hiPSCs and hESCs, could be considered in the design of future models.

Third, there is currently a relative paucity of reliable preclinical platforms that accurately predict the clinical efficacy of new drugs or of potential non-tumor-specific effects and toxicity. To address this knowledge gap, future studies should focus on generating more-integrative models that not only study the primary effect of tumor cells but also the interactions of these cells with their non-neoplastic microenvironment. Importantly, tumor models derived from human stem cells are, at least partly, developed in immunodeficient animals, which lack a complete immune system. The generation of stem cell-tumor models in humanized mice will, therefore, be integral to the design and implementation of effective clinical therapies. Humanized mice can be generated by transplanting human bone marrow to reconstitute the human immune complement (Walsh et al., 2017) or by using neuroimmune organoid models that incorporate human microglia and other microenvironment components prior to xenotransplantation in immunodeficient mice (Schafer et al., 2023). In the era of cancer immunotherapy, such models have already emerged for high-grade brain tumors (Chen et al., 2022; Choe et al., 2021; Salim et al., 2020; Vora et al., 2020) and other somatic cancers (Arenas et al., 2021; Hama et al., 2022; Martinez-Sabadell et al., 2022). Using these humanized mouse models provides an opportunity to address the unmet need of testing the efficiency of various immunotherapies.

Finally, as stem cell-based CNS tumor platforms are relatively novel, recently developed models have propelled the preclinical study of these difficult-to-treat diseases. However, there are still multiple challenges with regard to their immediate clinical application. Overcoming limitations in genetic, cellular and epigenetic diversity, which reflects the vast heterogeneity of the human condition, could accelerate the development of *ex vivo* or *in vivo* screening platforms for the discovery and testing of effective therapeutics to treat tumors of the human nervous system.
